# Combined Effect of Co-administration of Stromal Cell-Derived Factor-1 and Granulocyte-Colony Stimulating Factor on *Rat* Model of Alzheimer’s Disease

**DOI:** 10.3389/fnbeh.2022.796230

**Published:** 2022-03-02

**Authors:** Alireza Komaki, Siamak Shahidi, Nasrin Hashemi-Firouzi, Zahra Rafat, Arman Keymoradzadeh, Zoleikha Golipoor

**Affiliations:** ^1^Neurophysiology Research Center, Hamadan University of Medical Sciences, Hamadan, Iran; ^2^Department of Physiology, School of Medicine, Hamadan University of Medical Sciences, Hamadan, Iran; ^3^Department of Medical Microbiology, School of Medicine, Guilan University of Medical Sciences, Rasht, Iran; ^4^Student Research Committee, School of Medicine, Guilan University of Medical Sciences, Rasht, Iran; ^5^Neuroscience Research Center, Guilan University of Medical Sciences, Rasht, Iran

**Keywords:** Alzheimer’s disease, amyloid-beta, granulocyte colony-stimulating factor, memory, rat, stromal cell-derived factor-1

## Abstract

**Introduction:**

Alzheimer’s disease (AD) is a neurodegenerative disease that is characterized by amyloid plaque deposits, neuronal cell loss, and memory impairment. Granulocyte-colony stimulating factor (G-CSF) is a growth factor associated with AD improvement. Stromal cell-derived factor-1 (SDF-1) mediates therapeutic effects of G-CSF. This study investigated the effect of combination treatment of G-CSF and SDF-1 on amyloid plaque deposits, apoptosis, and behavior of AD rats.

**Methods:**

Intracerebroventricular amyloid-beta [Aβ(1-42)] peptide was used to induce AD in Aβ rats. There were six groups including naive control, sham-operated, Aβ, Aβ + G-CSF, Aβ + SDF-1, and Aβ + G-CSF + SDF-1. SDF-1 intra-cerebroventricular (ICV), G-CSF Subcutaneous (SC), or a combination of them were administered to Aβ rats weekly for 2 months. The cognition and memory were assessed using the novel object recognition, passive avoidance, and Morris water maze tests. Next, rat brains were removed and the amyloid plaque and apoptosis were detected in the brain and hippocampus using immunohistochemistry and TUNEL assay, respectively.

**Results:**

The amyloid-beta and apoptotic cell levels dropped in groups receiving SDF-1 and G-CSF combination compared to the Aβ group. Also, number of microglial cells increased significantly in the combination group compared to other treatment groups. Moreover, learning and memory were significantly improved in the combination group compared to the Aβ groups (*P* < 0.05).

**Conclusion:**

SDF-1 and G-CSF combination therapy can offer a promising strategy for AD.

## Introduction

Alzheimer’s disease (AD) is one of the progressive neurodegenerative diseases (Hardy and Selkoe, 2002). AD is characterized by amyloid plaque deposits and neuronal loss in the hippocampus and cortex, which lead to learning impairment and memory loss (Lazarov and Marr, 2010). Hippocampus is the main brain region involved in plasticity, learning, and memory (Mu and Gage, 2011). The production of new neurons is associated with learning and memory in the hippocampus, which is damaged at the early stages of AD (Lazarov and Marr, 2010). The stimulation of neurogenesis by stem cells presents a new treatment strategy for neurodegenerative diseases (Radad et al., 2017).

There is extensive research on the effect of stem cell transplantation for the treatment of lost or defective cells in AD (Phinney and Pittenger, 2017). Non-neural stem cells found in body tissues can be differentiated into one or more cell types (Atala et al., 2018). The transplantation of manipulated or differentiated stem cells is one of the greatest achievements of medical science and the role of these cells in tissue repair is astounding (Vasic et al., 2019). Today, scientists strive to stimulate body stem cells to cure neurological diseases with limited side effects (Shin et al., 2011). In the current era, scientists are investigating ways to use stem cells to treat many neurological diseases, including Alzheimer’s, with fewer side effects. As an example, in the mobilization of stem cells method, the patient’s stem cells moved to the lesion area and then used for healing and treatment (Wang et al., 2012). To treat neurological diseases using stem cells, SDF-1 can effectively induce stem cell mobilization (Wang et al., 2012).

Stromal cell-derived factor-1 (SDF-1) is a secreted protein that binds chemokine receptor type 4 (CXCR4), a seven-pass G protein-coupled membrane receptor (Molyneaux et al., 2003). SDF-1 has an important role in stem cell migration and proliferation (Guo et al., 2020). SDF-1 mediates the therapeutic effect of G-CSF (Wu et al., 2017). SDF-1 binding activates multiple signaling pathways involved in adhesion and migration (Kucia et al., 2004). It also decreases β-amyloid deposition (Sanchez-Ramos et al., 2009), and neuronal apoptosis (Guo et al., 2020) by enhancing mesenchymal stromal cell migration (Guo et al., 2020), and promoting the transfer of microglia from the peripheral blood to the central nerves system (Sanchez-Ramos et al., 2009). SDF-1 mediates its effect through CXCR4 (Kucia et al., 2004; Kowalski et al., 2017; Guo et al., 2020).

On the other hand, the first cytokine identified and rapidly transitioned into clinical medicine was granulocyte colony-stimulating factor (G-CSF) (Demetri and Griffin, 1991). G-CSF is able to mobilize hematopoietic stem cells from the bone marrow into the blood, which changed the face of hematopoietic stem cell transplantation (Wang et al., 2012). There is an inverse correlation between G-CSF and amyloid-beta (Aβ) in AD (Laske et al., 2009). The G-CSF induces neurotrophic activities in neurodegenerative diseases, moving the stem cell toward the injury site (Prakash et al., 2013). G-CSF is useful in inhibiting inflammatory cytokine expression, as inflammatory cytokines give rise to secondary damage (Prakash et al., 2013). The neuroprotective effect of G-CSF following cerebral ischemia has been recently reported (Kawas and Corrada, 2006). It is a neuronal ligand that can counteract programmed cell death (Kawada et al., 2006), decrease brain amyloid burden, and reverse cognitive impairment in the AD model (Wu et al., 2017; Spangenberg et al., 2019).

The G-CSF is another way to mobilize stem cells (Wu et al., 2017). This factor acts as a cytokine and promotes the survival, proliferation, and differentiation of bone marrow cells, and stimulation of circulating stem cells (Prakash et al., 2013). Experimental studies have shown that G-CSF triggers neurotrophic activity in neurodegenerative diseases and increases the movement of stem cells toward the damaged area (Sanchez-Ramos et al., 2009; Prakash et al., 2013; Guo et al., 2020). In addition, G-CSF decreases inflammation damage by inhibiting the expression of inflammatory cytokine (Prakash et al., 2013).

According to the above findings, it seems that both SDF-1 and G-CSF stimulate stem cell mobilization to the injury area. However, the combined treatment effects of SDF-1 and G-CSF have not been investigated in AD. The simultaneous use of SDF-1 and G-CSF strengthens the anti-apoptotic effect and decreases the amyloid plaque in the brain. In light of the findings, the aim of this study was to investigate the simultaneous effect of SDF-1 and G-CSF in the rat model of AD.

## Materials and Methods

### Animals

Forty-eight male Wistar rats weighing 250–300 g were procured from the Medical Faculty of Hamadan University of Medical Sciences. Rats were placed under standard lighting conditions (12-h light-dark cycles), temperature (22°C), and humidity (55–65%). Animals had *ad libitum* access to food and water for 1 week for acclimatization to the new environment. All procedures were performed in accordance with ethical codes stipulated by the Ethical Committee of Hamadan University of Medical Sciences (IR.UMSH.REC.1394.471) as well as the institutional and national guidelines regarding animal care and use.

### Drugs and Chemicals

Ketamine was purchased from Rotex Medical, Germany. Xylazine was bought from Alfasan, Woerden, Holland, and Aβ(1-42) was obtained from Tocris Bioscience, United Kingdom.

Stromal cell-derived factor-1 was purchased from (Sigma, SRP2252). SDF-1 powder (10 μg) was dissolved in 1 ml of phosphate buffered saline (PBS). Then, the stock solution was diluted 1:20 to obtain a solution of 500 ng/ml and was injected intraventricular once a week for 2 months (Zendedel et al., 2016).

Human G-CSF manufactured by pharmacy (Pooyesh Darou, Tehran, Iran). The standard dose of G-CSF was injected SC once a week for 2 months at a concentration of 100 g/kg (Takeuchi et al., 2007; Song et al., 2016).

### Surgery and Injection of Amyloid-Beta (1-42)

The surgery was performed as described in previous studies (Nasiri et al., 2019). Briefly, the rats were anesthetized with a mixer of xylazine and ketamine (10/100 mg/kg). Then, the animals were submitted to a stereotaxic apparatus (Stoelting, United States). The intra-cerebroventricular (ICV) coordinates for Aβ injection matched the rat brain in stereotaxic [AP: 1.2 mm, ML: 2 mm, and DV: 4 mm from Bregma (Paxinos and Watson, 1998)]. The Aβ solution (5 μg/μL) was bilaterally injected into that area. The skin was sutured and it took animals 1 week to recover.

#### Intraventricular Catheter Insertion

There are several methods for inducing memory impairment in animal models, including intracerebroventricular injections of beta-amyloid. In the present study, for induction of memory impairment model, the animals were first anesthetized with ketamine (100 mg/kg) and xylazine (10 mg/kg) and transferred to a stereotaxic device. After observing the ocular reflex, stereotaxic rods were placed inside the animal’s ear, and the head was fixed in the device in the middle area. Then, the shaved part of the scalp is removed with scissors in the desired location. Following this, Bregma and Lambda regions were identified based on the Paxinos atlas and the distance between these two points was determined. Then, the following coordinates were recorded: AP: 0.9 mm (Anterior posterior), ML: 1.5 mm (Medial lateral), DV: 3.2 mm (Ventricle Depth). Then, the right side of the brain was pierced using a dental drill. Following that, a 23-gauge cannula fixture (12-millimeter length) was lowered to a depth of 3 mm from the surface of the head bone and then attached to the head using dental cement and a screw. Finally, through the fixed cannula on the head, beta-amyloid or SDF-1 were injected using the Hamilton syringe.

### Experimental Design

**FIGURE 1:**
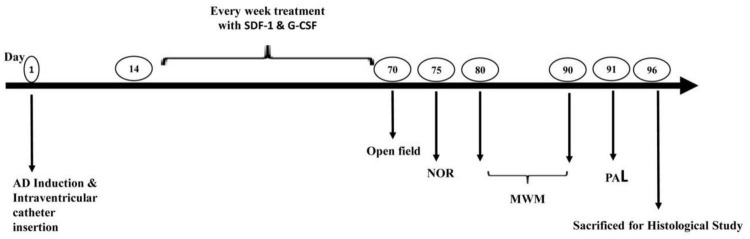
Study design diagram. Alzheimer’s was induced in the rat model of Alzheimer’s disease by ICV injection of Aβ. The administration of SDF-1 and G-CSF was performed every week for 2 months after the injections of Aβ. Finally, behavioral studies (Open field, NOR Test, MWM, and PAL test) were performed and brain tissues were studied histologically.

Intra-ventricular injection of Aβ(1-42) was used to induce AD. Animals were randomized into six groups (*n* = 8) before the operation. The control group did not receive any treatment or surgery. The sham group underwent surgery without Aβ injection. Aβ group received Aβ-peptide without any treatment. The SDF-1 group received SDF-1 through intravenous administration 2 weeks after Aβ-peptide injection for a period of 2 months. Also, G-CSF group received G-CSF via subcutaneous administration 2 weeks after Aβ-peptide injection for a period of 2 months. G-CSF and SDF-1 group received both SDF-1 and G-CSF administration. The SDF-1 and G-CSF were administrated every week for 2 months. At the end of treatment, the memory of animals was assessed by behavioral tests and histological studies, which were performed after sacrificing animals (Figure 1).

#### Behavioral Studies

##### Open-Field Test

The open-field test evaluated animals’ locomotor activities. The apparatus was made of a brown wooden field (surface area: 50 × 50 cm and wall height: 38 cm) with low ambient lighting. The rats were first placed in the open field, and allowed to explore the surrounding area for 10 min. The velocity and total distance traveled were recorded by a camera (Nasiri et al., 2019).

##### Novel Object Recognition Test

A wooden open box (35 × 43 × 40 cm) was used as the apparatus of a novel object recognition test. The animals were habituated in the empty box for 5 min. After 2 h, two similar objects were presented to rats for 5 min in a learning trial. On the next day, the testing phase was performed with one of familiar objects and a novel object for 5 min. The ratio of time spent on the novel object to the total time spent on each object was calculated as the distinction index. After each session, all areas and objects were cleaned and any residual odors was removed by 70% ethanol.

##### Morris Water Maze Test

The spatial memory was evaluated by Morris water maze (MWM) test. Briefly, a black circular pool (155 cm in diameter, 60 cm in height) was filled with water (22 ± 1°C) to a depth of 40 cm. The hidden black escape platform was placed 2 cm under the water surface on the northern quadrant of the pool. The shapes on the walls served as visual cues to rats in order to find the hidden platform. The learning trials consisted of two blocks of four trials per day for four consecutive days. The time needed to reach the hidden platform was recorded during the learning phase. One day after the last session, the retention (probe) test was carried out without the escape platform. The rats were placed into water and the duration of swimming in the target quadrant was recorded for 60 s.

#### Passive Avoidance Learning

The transparent plastic apparatus (20 cm × 20 cm × 30 cm dimensions) consisted of light and dark compartments with a stainless-steel rod (3 mm in diameter) on the floor. An opaque guillotine door (6 cm × 8 cm) was placed between the two compartments. The electricity in the dark compartment floor was induced a shock generator (Behbood Pardaz Co., Tehran, Iran).

##### Training

Initially, the apparatus was presented to each rat in two trials. To do so, the rats were placed in the light compartment away from the door for 5 s. Then, the guillotine door was raised. The rats headed for the dark environment instinctively. When they moved into the dark compartment, the guillotine door was closed and the rats remained in this compartment for 30 s. This trial was repeated with 30 min intervals. In another scenario, after inserting rats in the dark compartment, we measured the delay in entering the dark compartment or step-through latency (STLa). Following the spontaneous entry of rats into the dark compartment, the guillotine door was shut for 30 s and an electrical shock was applied (0.5 mA, 3 s). The procedure was repeated after 2 min. The training was halted when animals managed to stay in the light compartment for 120 consecutive seconds. We recorded the number of trials that involved entering the dark compartment.

##### Retention Test

For the purpose of the retention test, the rats were placed inside the light compartment for 5 s. Then, the guillotine door was shut and the step-through latencies in retention (STLr) was recorded in the retention trial. The amount of time spent in the dark compartment (TDC) was up to 5 min. The retention test was terminated when a rat failed to enter the dark chamber within 3 s (Hasanein and Shahidi, 2011).

#### Histological and Immunohistochemistry Studies

The animals were sacrificed 69 days after the study, and brain tissues were stained by fluorescent antibodies against Aβ(1-42), and Ionized calcium-binding adaptor molecule 1 (IBA1). Animals were deeply anesthetized with ketamine and xylazine and perfused with 150–200 ml PBS and 4% paraformaldehyde. Brain tissues were then harvested and embedded in paraffin (Merck, Germany) and 5 μm paraffin sections were microtomed. A total of three brain were selected to count beta-amyloid plaques and microglia cells. From each animal, five sections were analyzed with the conditions mentioned. A section with a thickness of five microns was prepared from every 20 tissue sections to examine beta-amyloid and microglia.

For immunohistochemistry, the paraffin sections were gradually dewaxed into the water, immersed in 0.01 mol/L citrate buffers and microwaved for the antigen retrieval. Afterward, the sections were incubated with antibodies against Aβ(1-42) (ORB10087) and Iba1 (ORB336635) overnight at 4°C. Sections were then washed with PBS and incubated with the secondary fluorescent antibodies (anti-rabbit, 406403) for 2 h at room temperature. Sections were washed with PBS, and nuclei were counterstained with DAPI (the chromogen) for 45 min. Images were captured by a fluorescence microscope (Leica AF 6000). In this study, fluorescent microscopy and ImageJ software were used to observe Beta amyloid plaques and Iba1 cells directly. Counting beta-amyloid plaques and Iba1 cells were done after merging their nuclei with DAPI.

#### Apoptosis (Terminal Deoxynucleotidyl Transferase dUTP Nick End Labeling Assay)

Terminal deoxynucleotidyl transferase dUTP nick end labeling (TUNEL) is a known method of detecting DNA fragments that is used to identify apoptotic cells. After the behavioral test, the rats were intracardially perfused with saline followed by 4% paraformaldehyde in 0.1 M phosphate buffer (pH = 7.4), and their brains were fixed. To measure apoptosis, first three animals were selected, and then five tissues with a thickness of 5 microns from each animal were prepared.

In the next step, the fixed brains were serially sectioned into 5-μm coronal sections by a microtome (Leitz GmBH, Wetzlar, Germany), hydrated, and prepared for the detection of DNA fragmentation and apoptotic cells using the TUNEL kit (Roche), as described earlier. Following the permeabilization of samples (citrate buffer pH: 6), the sections were incubated using the TUNEL reaction mixture for 60 min at 37°C. The converter-peroxidase (30 min) and 3,3′-diaminobenzidine substrates (10 min) were added to samples. The sections were counterstained with hematoxylin and analyzed under a light microscope (400× magnification, Olympus). A cell with dark brown particles in the nucleus was defined as an apoptotic hippocampal neuron. For each animal, the mean number of apoptotic cells was obtained by counting five coronal sections.

#### Statistics

Data obtained from the open field and immunostaining tests were analyzed by one-way analysis of variance (ANOVA) test. Also, the Tukey’s *post hoc* test was used to compare the mean of all treatments to the mean of other treatments. *P-*values ≤ 0.05 was considered as statistically significant.

## Results

### Effect of Stromal Cell-Derived Factor-1 and Granulocyte-Colony Stimulating Factor on the Open Field Test

**FIGURE 2:**
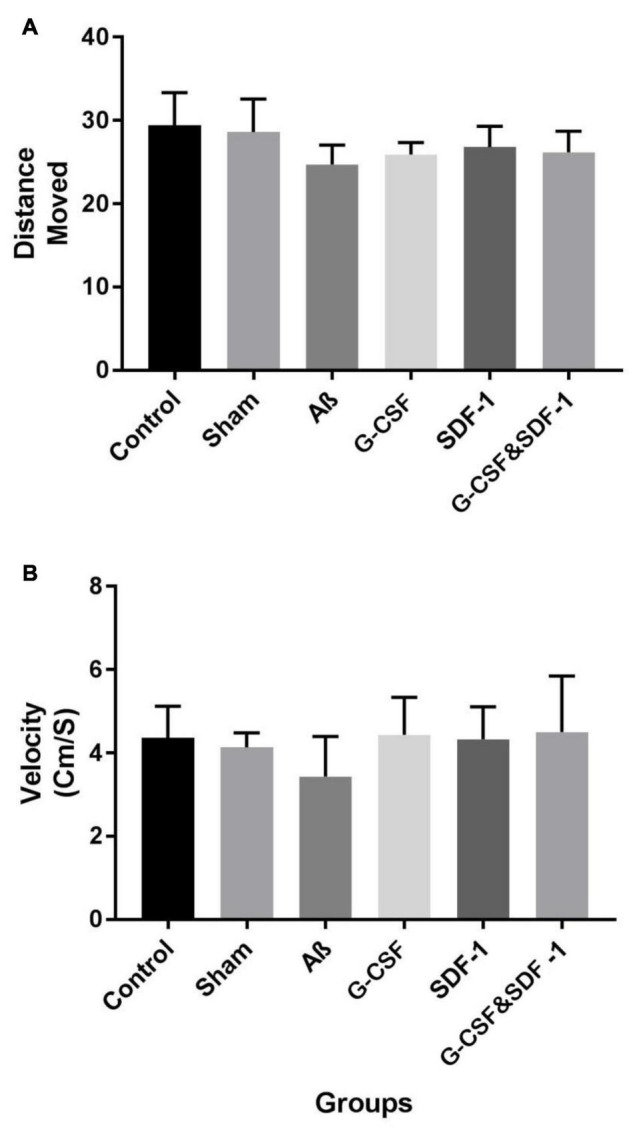
Effect of treatment in Aβ rat model on distance move **(A)** and velocity **(B)** in open field test. Data are represented as Mean ± SEM.

The results of the open field test by one-way ANOVA revealed that there was no significant difference between all groups in terms of distance traveled in the open field box (*P* < 0.001). Also, the groups were not significantly different in the movement of locomotion in the open field box (*P* < 0.01) (Figure 2).

### Effect of Stromal Cell-Derived Factor-1 and Granulocyte-Colony Stimulating Factor on the Novel Object Recognition Test

**FIGURE 3:**
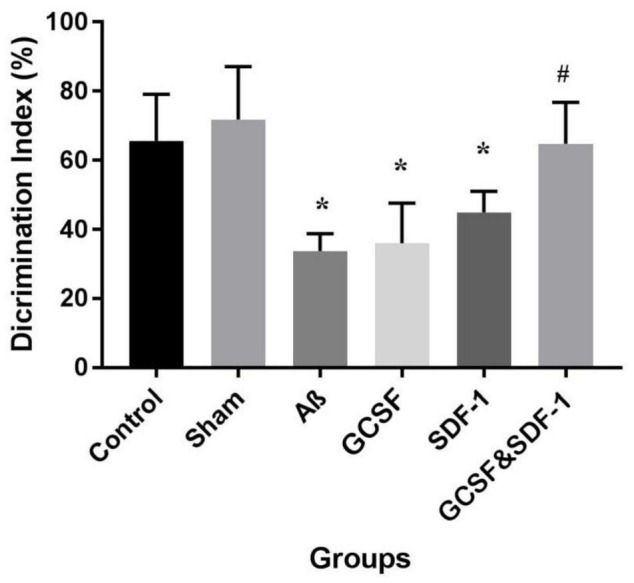
Effect of SDF-1 and G-CSF treatment on discrimination index in object recognition test. (*P < 0.05) as compared with control and sham group. (#*P* < 0.05) as compared with G-SDF and SDF-1 groups. Each column represents Mean ± SEM.

The result of NOR test and total discrimination index are shown in Figure 3. The one-way ANOVA analysis demonstrated the main difference between groups. Aβ, G-CSF, and SDF-1 groups had a lower discrimination index compared to control and sham groups (*P* < 0.05). The combined treatment with G-CSF and SDF-1 increased the discrimination index compared to the G-CSF and SDF-1 group (*P* < 0.01).

### Effect of Stromal Cell-Derived Factor-1 and Granulocyte-Colony Stimulating Factor on the Morris Water Maze Test

**FIGURE 4:**
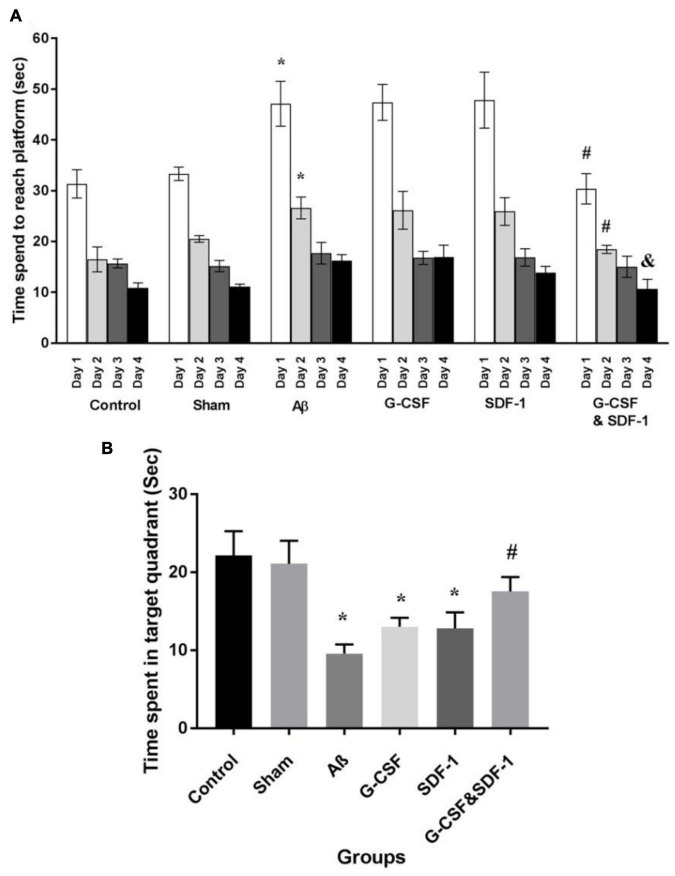
Effect of SDF-1 and G-CSF treatment in the Morris Water Maze test. **(A)** Time spend to find platform during learning days. **(B)** Time spend in target quadrant in test day. (*P < 0.05) as compared with control and sham groups. (#*P* < 0.05) as compared with G-SDF and SDF-1 groups. (&*P* < 0.05) Aβ group. Each column represents Mean ± SEM.

The results of the spatial learning phase of MWM are shown in Figure 4A. All animals learned the hidden platform after 4 days of learning. It took more time for Aβ, G-CSF, and SDF-1 groups to reach the platform compared to the control and sham animals (*P* < 0.05). The combined treatment with G-CSF and SDF-1 decreased the time required to find the platform compared to G-CSF, SDF-1, and Aβ groups (*P* < 0.05). Figure 4B shows the results of the probe test. The Aβ, G-CSF and SDF-1 groups spent less time on the target quadrant than control and sham groups did (*P* < 0.05). The animals treated with a combination of G-CSF and SDF-1 spent more time than Aβ group in the target quadrant (*P* < 0.05).

### Passive Avoidance Test Finding

**FIGURE 5:**
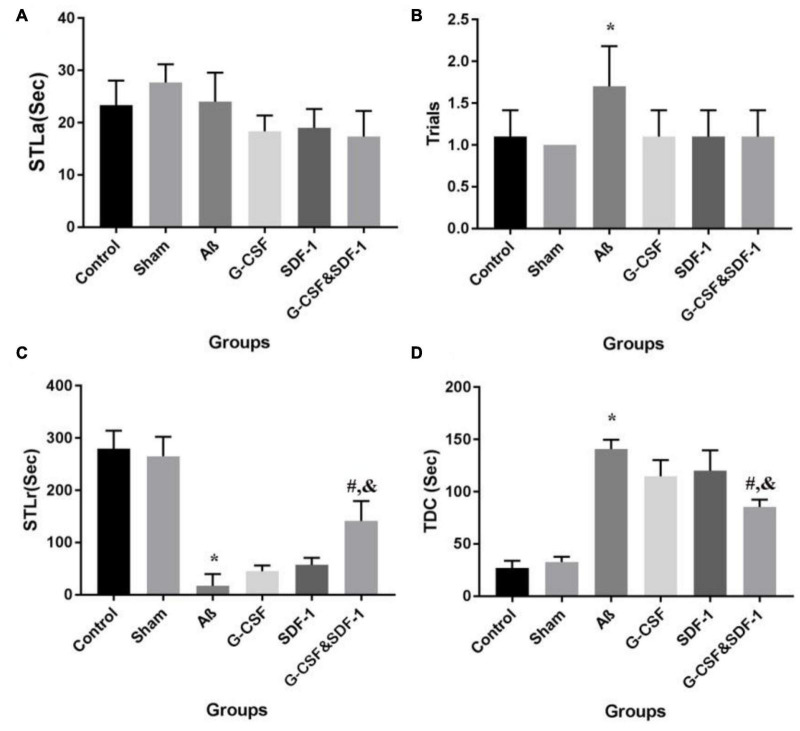
Effect of SDF-1 and G-CSF treatment on passive avoidance learning task **(A)**, number of trials to acquisition in acquisition **(B)**, step-through latency in retention **(C)**, time spent in the dark compartment **(D)**. (*P < 0.05) as compared with control group. (#*P* < 0.05) as compared with G-SDF and SDF-1 groups. (&*P* < 0.05) as compared with Aβ group. Each column represents Mean ± SEM.

Statistical analysis by ANOVA revealed that the two groups were not different in STLa (Figure 5A). The number of trials was different between groups. Aβ rats received more trials than control and sham groups did (*P* < 0.05; Figure 5B).

A significant difference in STLr time was revealed by ANOVA (Figure 5C). The STLr time was lower in Aβ rats than in control and sham groups (*P* < 0.05). The STLr time in the combined treatment with G-CSF and SDF-1 groups was higher than G-CSF and other single groups (*P* < 0.05). The one-way ANOVA showed differences between groups in TDC on the test day. The Aβ group spent more time on TDC than control and sham groups did (Figure 5D) (*P* < 0.05). The combined administration of SDF-1 and G-CSF decreased TDC time in treated rats compared to the Aβ group (*P* < 0.05).

### Histological and Immunohistochemistry Findings

#### Effect of Stromal Cell-Derived Factor-1 and Granulocyte-Colony Stimulating Factor on the Amount of Beta Amyloid Deposition

**FIGURE 6:**
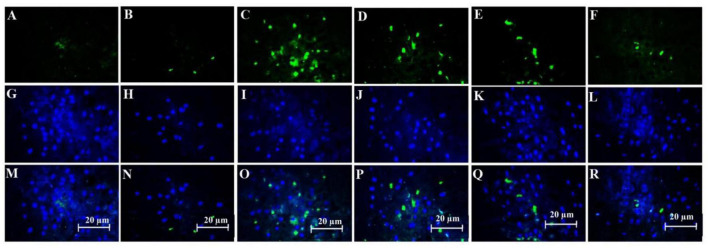
Effect of SDF-1 and G-CSF on the amount of anti-beta amyloid deposition in the cortex of all experimental groups: **(A)** (Control), **(B)** (Sham), **(C)** (βA), **(D)** (G-CSF), **(E)** (SDF-1), **(F)** (G-CSF&SDF-1); DAPI **(G–L)**, Merge **(M–R)**.

**FIGURE 7:**
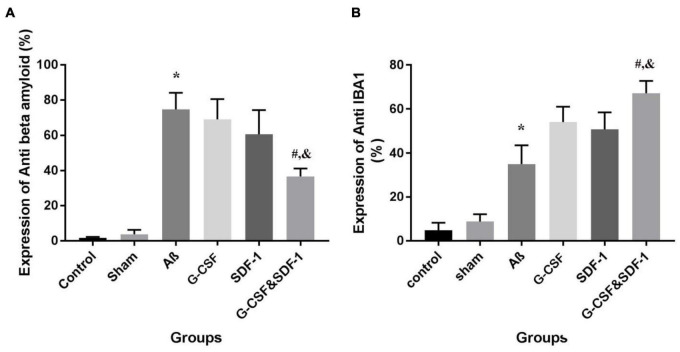
The expression percentage of anti-beta amyloid **(A)** and anti-IBA1 **(B)** in the brains of all six studies groups. (*P < 0.05) as compared with control and sham group. (#*P* < 0.05) as compared with G-SDF and SDF-1 groups. (&*P* < 0.05) as compared with Aβ.

The combination of SDF-1 and G-CSF significantly decreased amyloid plaque deposition in comparison with G-CSF and SDF-1 groups (Figures 6, 7A) (*P* < 0.05).

#### Effect of Stromal Cell-Derived Factor-1 and Granulocyte-Colony Stimulating Factor on the Number of Microglial Cells

**FIGURE 8:**
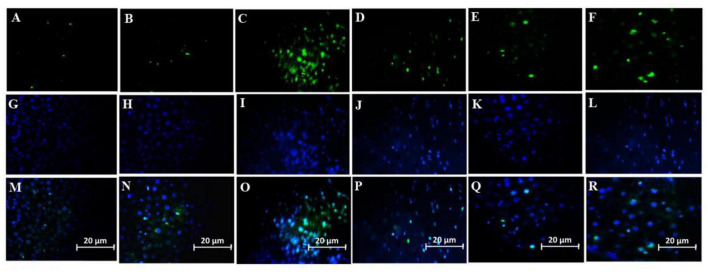
Effect of SDF-1 and G-CSF on the amount of anti-IBA1 deposition in the cortex of all experimental groups: **(A)** (Control), **(B)** (Sham), **(C)** (βA), **(D)** (G-CSF), **(E)** (SDF-1), **(F)** (G-CSF&SDF-1): DAPI **(G–L)**, Merge **(M–R)**.

Number of Microglial cells significantly increased in the group treated with a combination of SDF-1 and G-CSF compared to other treatment groups (Figures 7B, 8) (*P* < 0.05).

#### Effects Stromal Cell-Derived Factor-1 and Granulocyte-Colony Stimulating Factor on Hippocampal Apoptotic Cells

**FIGURE 9:**
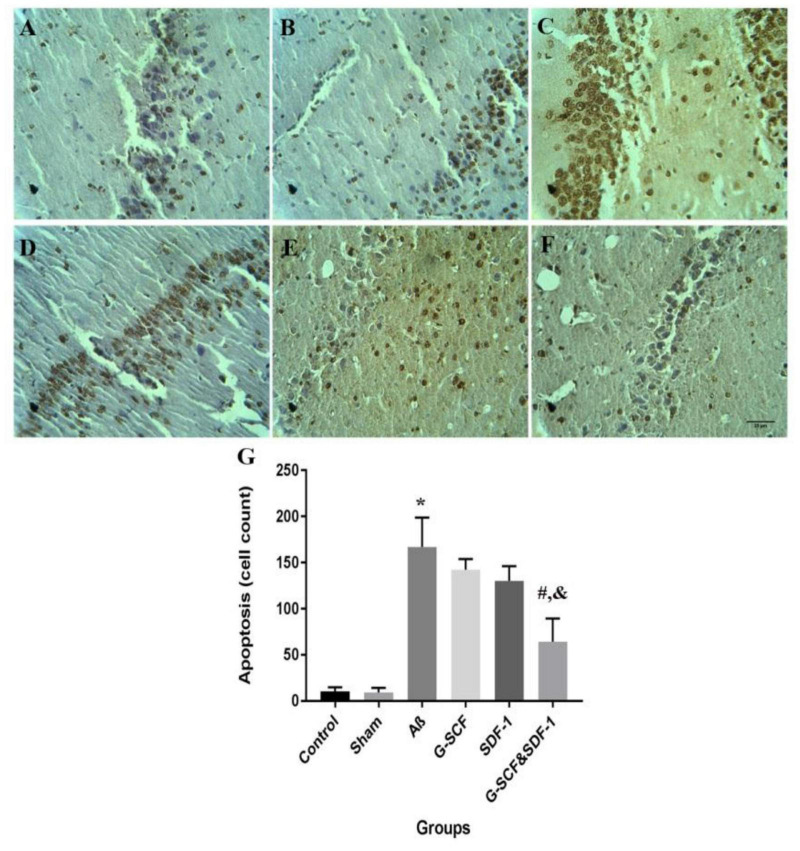
Light micrographs illustrated the apoptosis cell in the CA1 region of hippocampal. **(A)** Sections derived from the **(A)** control; **(B)** sham; **(C)** Aβ; **(D)** G-CSF; **(E)** SDF-1; and **(F)** SDF-1&G-CSF groups. **(G)** The mean of apoptotic cell count was calculated. (*P < 0.01) as compared with control group. (#*P* < 0.05) as compared with G-SDF and SDF-1 groups. (&*P* < 0.05) as compared with Aβ. Each column represents Mean ± SEM.

Figures 9A–F shows neurons in the CA1 area of hippocampus in the experimental groups. The TUNEL staining identified apoptotic neurons in the sections. One-way ANOVA mirrored a significant difference in the number of apoptotic cells between groups (Figure 9G). Tukey’s *post hoc* test showed that the number of apoptotic cells in the Aβ group was significantly higher than in the control and sham groups (*P* < 0.001). The number of apoptotic cells was significantly lower in the combined treatment with SDF-1 and G-CSF group than in the Aβ group (*P* < 0.05). There was, however, no significant difference in the number of apoptotic cells between the control and sham groups.

## Discussion

This study investigated the combined treatment effects of SDF-1 and G-CSF on rats receiving ICV injection of beta-amyloid (1-42) peptide over 2 months. The results of behavioral tests show that Aβ provoked learning deficiency whilst the combined treatment with SDF-1 and G-CSF resolved this impairment. In addition, the SDF-1 and G-CSF treatment improved reacquisition and memory in the combination group. These findings suggest that both spatial and avoidance memory impairment induced by Aβ peptide could be resolved by combined administration of SDF-1 and G-CSF. In the present study, 96 days after Aβ injection, the amount of beta-amyloid deposition, microglia, and cell death was assessed in the brains of rats in all groups using the immunohistochemical method. The results showed that combined injection of SDF-1 and G-CSF decreased the beta-amyloid accumulations and apoptosis in the hippocampus. Also, the combined treatment of SDF-1 and G-CSF increased microglia in the brain.

Stromal cell-derived factor-1 is one of the chemokines of the CXC family that stimulates and implants bone marrow cells through the CXCR4 receptor (Guo et al., 2015; Zhou et al., 2019). The binding of SDF-1 to CXCR4 modulates several biological reactions in CXCR4^+^ cells, such as cell growth, proliferation, angiogenesis, and cell survival (Moepps et al., 2000; Kucia et al., 2004; Kowalski et al., 2017). Transgenic mice with abnormalities in the SDF-1 and CXCR4 phenotypes have defective central nervous system (Moepps et al., 2000). SDF-1 is secreted by microglia, astrocytes, and neurons in the central nervous system (Bajetto et al., 1999). A great amount of SDF-1 is secreted by activated glial cells under pathological conditions (Hill et al., 2004). In line with our findings, the results of another study showed a surge in microglia and memory improvement in the groups treated with SDF-1 and G-CSF (Shin et al., 2011). The increase in microglia indicates that SDF-1 is an effective adjuvant in inducing migration into brain (Shin et al., 2011). It also decreased the level of amyloid-beta (1-42) (Laske et al., 2009; Sanchez-Ramos et al., 2009) and neuronal apoptosis by enhancing mesenchymal stromal cell migration in the brain (Guo et al., 2020).

On the other hand, G-CSF stimulates the mobilization of bone marrow-derived microglia into the brain (Sanchez-Ramos et al., 2008) and is essential for microglia survival (Spangenberg et al., 2019). G-CSF affects microglia increase by two mechanisms. In the present study, the ratio of microglia in the rats receiving combined SDF-1 and G-CSF treatment was higher than the G-CSF group. This finding reflects the synergistic activity of SDF-1 and G-CSF (Hasselblatt et al., 2007; Sanchez-Ramos et al., 2009). G-CSF treatment increased neurogenesis and mobilization of the bone marrow mesenchymal stem cells in the brain (Wu et al., 2017).

Oxidative stress and cell death are major side effects of AD (Hardy and Selkoe, 2002; Gella and Durany, 2009). The amyloid-beta accumulation reduces the cellular respiration in neurons and astrocyte cells (Rhein et al., 2009). Irregular reactions in the electron transfer chain increase the free radicals production, alter the mitochondrial permeability transfer (Pickett et al., 2018), induce mitochondrial fragmentation and lead to neuronal cell death (Dowding et al., 2014).

Furthermore, the study showed that combined treatment with G-CSF and SDF-1 had a reinforced effect in on apoptosis inhibition in AD. Recently, evidence suggests that G-CSF plays a protective role in the brain by enhancing anti-apoptotic and anti-inflammatory effects (Huang et al., 2017). This suggests that G-CSF attenuates neuronal apoptosis and cell death in the brain (Strecker et al., 2010; Huang et al., 2017). G-CSF activated anti-inflammatory actions and survival signaling mechanisms, which triggered angiogenesis (Solaroglu et al., 2009), and anti-apoptotic activities in the neurons (Komine-Kobayashi et al., 2006). G-CSF attenuates apoptosis and enhances angiogenesis in the brain (Huang et al., 2017). In addition, SDF-1 has an anti-apoptotic activity and protects cells against stressful situations (Lataillade et al., 2002). The bone marrow-derived stem cells activated the SDF-1/CXCR4 axis and reduced neuronal apoptosis in the injured brain (Guo et al., 2020). SDF-1 is the endogenous self-repair mechanism and it is facilitate repair-competent cells to the injured region of brain in AD. G-CSF treatment mobilizes the bone marrow derived stromal stem cells (BMMSCs) and potentiate repair-competent cells in the ameliorating mechanism (Song et al., 2016). The increases concentration of G-CSF and SDF-1 in the peripheral blood is associated with increased number of hematopoietic stem cells (HSCs) into the peripheral bloodstream. There is strong correlation between serum G-CSF concentration and SDF-1 (Gębura et al., 2019) and G-CSF concentration involved in release of HSC (Gębura et al., 2019).

## Conclusion

The combined treatment with G-CSF and SDF-1 can be a rich source of cells for AD treatment. Both G-CSF and SDF-1 bolstered cell migration to the brain. The stem cells are able to migrate from the peripheral blood and proliferate in the presence of SDF-1 and G-CSF. The combined treatment with SDF-1 and G-CSF has huge potentials for tissue regeneration. However, the present study did not measure the level of SDF-1 and G-CSF in the brain. The study of stem cell migration and implantation shed further light on AD treatment.

## Data Availability Statement

The original contributions presented in the study are included in the article/supplementary material, further inquiries can be directed to the corresponding author/s.

## Ethics Statement

The animal study was reviewed and approved by the Ethical Committee of Hamadan University of Medical Sciences (IR.UMSH.REC.1394.471).

## Author Contributions

AKo and ZG designed and managed the study. SS and NH-F performed the experiments, collected the data, and wrote the manuscript. ZR and AKe assisted in experiments. All authors read and approved the final manuscript.

## Conflict of Interest

The authors declare that the research was conducted in the absence of any commercial or financial relationships that could be construed as a potential conflict of interest.

## Publisher’s Note

All claims expressed in this article are solely those of the authors and do not necessarily represent those of their affiliated organizations, or those of the publisher, the editors and the reviewers. Any product that may be evaluated in this article, or claim that may be made by its manufacturer, is not guaranteed or endorsed by the publisher.
